# ZDHHC11B is decreased in lung adenocarcinoma and inhibits tumorigenesis via regulating epithelial–mesenchymal transition

**DOI:** 10.1002/cam4.6345

**Published:** 2023-07-11

**Authors:** Huanyu Dai, Ruiyue Wu, Jiatong Zhang, Rong Dou, Maohong Xu, Jiahui Wang, Jun Wang, Fei Su, Tao Zhang

**Affiliations:** ^1^ Department of Oncology The First Hospital of Lanzhou University Lanzhou China; ^2^ The First Clinical Medical College Lanzhou University Lanzhou China; ^3^ The Second Clinical Medical College Lanzhou University Lanzhou China; ^4^ The College of Pharmacy Lanzhou University Lanzhou China

**Keywords:** biomarkers, epithelial–mesenchymal transition, lung adenocarcinoma, tumorigenesis, ZDHHC11B

## Abstract

**Purpose:**

The role and mechanism of zinc finger DHHC protein 11B (ZDHHC11B) in lung adenocarcinoma (LUAD) remain unclear. We, thus, analyzed the expression pattern, biological function, and potential mechanism of ZDHHC11B in LUAD.

**Methods:**

The expression level and prognostic value of ZDHHC11B were evaluated based on The Cancer Genome Atlas (TCGA) database and further confirmed in LUAD tissues and cells. The effect of ZDHHC11B on the malignant biological progression of LUAD was evaluated in vitro and in vivo. Gene set enrichment analysis (GSEA) and western blot were used to explore the molecular mechanisms of ZDHHC11B.

**Results:**

In vitro, ZDHHC11B inhibited the proliferation, migration, and invasion of LUAD cells and induced the apoptosis of LUAD cells. In addition, ZDHHC11B inhibited the growth of tumors in nude mice. GSEA revealed that ZDHHC11B expression is positively correlated with epithelial–mesenchymal transition (EMT). Western blot analysis demonstrated that molecular markers of EMT were inhibited under ZDHHC11B overexpression conditions.

**Conclusions:**

Our findings indicated that ZDHHC11B plays a significant role in inhibiting tumorigenesis via EMT. In addition, ZDHHC11B may be a candidate molecular target for LUAD treatment.

## 1 INTRODUCTION

Lung cancer has the highest morbidity and mortality rate and has become a major public health problem worldwide.^1^, ^2^ Lung adenocarcinoma (LUAD) is the most common pathological subtype in all lung cancers. The presence of lymph node metastasis is a crucial factor that impacts the long‐term survival of individuals with lung cancer who have undergone surgery.^3^ The metastasis rate of lymph nodes is also determined by the degree of differentiation of LUAD.^4^, ^5^ Although new drugs and treatment modalities have improved outcomes for patients with LUAD, the comprehensive management of LUAD still faces great challenges. It is still the focus of current research to actively explore the molecular mechanism and seek new therapeutic targets in LUAD.

The zinc finger DHHC proteins (ZDHHC) family has *S*‐acyltransferase activity. These enzymes are commonly found in the eukarya domain, and there are 23 distinct ZDHHC‐encoding genes in the human genome. ZDHHC enzymes mediate a large number of intracellular *S*‐acylation (also known as palmitoylation) reactions that reversibly attach fatty acids to cysteine residues, thereby affecting the function of a wide range of proteins.^6^ One of the effects of *S*‐acylation mediated by ZDHHC enzymes is an enhancement in the membrane attachment capacity of soluble proteins, regulating the intracellular spatial structure of proteins, modulating protein assembly and stability, and affecting protein expression at the membrane.^7^, ^8^, ^9^ These regulatory mechanisms often affect many important molecular structures in the body, such as G proteins, ion channels, and membrane receptors.^10^ Thus, the ZDHHC family is essential for normal cellular physiology, and there is growing evidence that its dysfunction is associated with specific disease processes.^11^, ^12^ Therefore, regulation of the ZDHHC protein family is receiving increasing attention as a potential therapeutic target.

ZDHHC family is a category of enzymes involved in post‐translational modification through *S*‐acyltransferase activity, which can regulate the function of downstream proteins by altering the localization and function of proteins, especially lipoproteins, such as Hedgehog, Wnt, and RAS.^13^, ^14^ Therefore, ZDHHCs family is not only involved in regulation of normal cells but also plays an important role in the growth of tumor cells.^15^ Recent research has identified a link between ZDHHCs and several types of cancer. For instance, ZDHHC1 has been associated with endometrial, renal, and pancreatic cancers, while ZDHHC3 has been linked to breast carcinoma and ZDHHC9 with cervical cancer. Among these studies, high expression of ZDHHC18 was found to be an unfavorable prognostic factor for liver cancer and glioma.^16^ In LUAD studies, the ZDHHC protein family was found to still play an important role.^17^ In this study, we aimed to perform a comprehensive analysis of 23 ZDHHCs family members to explore the impact of each protein in LUAD through a bioinformatics approach and verify its functionality in vitro and in vivo experiments.

## 2 MATERIALS AND METHODS

### 2.1 Data collection and analysis of LUAD

The mRNA expression and clinical data from 526 LUAD samples and 59 normal samples were obtained from The Cancer Genome Atlas (TCGA) database. The differentially expressed genes (DEGs) of the ZDHHCs were identified using the Limma version 4.0.2 R package. Univariate Cox regression analysis was performed to identify the prognostic genes. Kaplan–Meier analysis was used to assess the survival benefit of ZDHHC11B in LUAD.

### 2.2 Tissue collection and cell culture

This study was approved by the Ethics Committee of The First Hospital of Lanzhou University. Fifteen pairs of LUAD tissues and matched lung tissues were collected from patients in The First Hospital of Lanzhou University. All study procedures were performed according to the Declaration of Helsinki ethical principles. Informed consent was obtained from the patients. The 16HBE, A549, H460, H1299, and H292 cell lines were purchased from the National Infrastructure of Cell Line Resource (Shanghai). All cell lines were cultivated in RPMI 1640 containing 10% fetal bovine serum, penicillin (100 U/mL), streptomycin (0.1 mg/mL), and cultured in an incubator at 37°C with 5% CO_2_.

### 2.3 Immunohistochemistry

For the pathological evaluation of samples, both LUAD and normal tissues embedded in paraffin were first stained with hematoxylin and eosin (H&E) and then cut into slices. After dewaxing, rehydration, and incubation with 90% formic acid, the sections were blocked with 1% BAS and incubated with anti‐ZDHHC11B antibody (1:200, Invitrogen) overnight. On the next day, the samples were washed with phosphate buffer saline and incubated with horseradish peroxidase‐labeled secondary antibodies. Diaminobenzidine and 20% hematoxylin were used to stain the sections. We used an Olympus light microscope to capture the images.

### 2.4 Western blot

LUAD tissues and cells were lysed with the presence of protease inhibitors. Protein concentration was assessed using a BCA kit (Solarbio). Proteins were electrophoresed on a 10% SDS‐PAGE and transferred onto a PVDF membrane, which was blocked with 5% skim milk for 1 h at room temperature. Primary antibodies, including anti‐GAPDH (1:10000; Proteintech), anti‐ZDHHC11B (1:800, Invitrogen), anti‐Snail (1:1000, Proteintech), anti‐E‐cadherin (1:1000, Proteintech), anti‐N‐cadherin (1:1000, Proteintech), anti‐MMP9 (1:1500, Proteintech), and anti‐vimentin (1:800, Proteintech), were added to the membrane and incubated overnight at 4°C. The samples were incubated with secondary antibodies. Chemiluminescence was detected using the Tanon 4600 imaging system.

### 2.5 Cell transfection

The ZDHHC11B sequence was subcloned into pcDNA3.1 for lentivirus synthesis using GenePharma (Shanghai). The empty vector was used in the control group. The lentivirus was transfected into the A549 and H292 cells in the presence of polybrene. Small hairpin RNA (shRNA) targeting ZDHHC11B were synthesized by GenePharma. The target sequences of ZDHHC11B shRNAs are provided in Table S1. The stable cells were selected using puromycin.

### 2.6 Cell proliferation assays

CCK‐8 (Solarbio) was used to detect cell growth. The cells were plated in 96‐well plates at a density of 1000 cells/well. Then, 10 μL of CCK‐8 was added to each well every 24 h for 3 days according to the manufacturer's protocol. Absorbance was measured at 450 nm using a microplate reader. For the 5‐ethynyl‐2′‐deoxyuridine (EdU) assay, cells fixed with 4% formaldehyde solution were stained with an EdU kit (RiboBio) according to the manufacturer's protocol.

### 2.7 TUNEL assay

The TUNEL kit (Solarbio) can be used to detect DNA fragmentation, a feature of apoptotic cells. An apoptosis assay was performed according to the protocol provided by the manufacturer of the TUNEL kit. An Olympus microscope was used to collect the images, and TUNEL‐positive cells were counted using the Image J software.

### 2.8 Transwell assay

For cell invasion, Matrigel‐coated Transwell chambers (Corning) were used. A complete medium was added to the lower chamber, and a serum‐free medium was added to the upper chamber. Invading cells were fixed and stained with a crystal violet solution for counting. The cell migration assay was performed without Matrigel coating. The number of migrated or invaded cells was measured under a microscope.

### 2.9 Xenograft model

A549 cells were prepared for the construction of a model that stably overexpressed either ZDHHC11B or the control vector. Nude male mice (*n* = 12) were divided into two groups. Each group was separately injected subcutaneously with 5 × 10^5^ cells. Tumor volume was measured weekly. After 4 weeks, the animals were euthanized, and the tumors were removed and weighed. The animal experiments were approved by the Ethics Committee of The First Hospital of Lanzhou University.

### 2.10 Gene set enrichment analysis (GSEA)

To explore the signaling pathways associated with ZDHHC11B expression in LUAD, GSEA was conducted using TCGA database. Enrichment analysis was performed using gene sets downloaded from the Molecular Signatures Database (MsigDB, http://software.broadinstitute.org/gsea/index.jsp).

### 2.11 Statistical analysis

The R (v.4.0.2) software was used for statistical analyses. Cox regression analysis was used to evaluate the influence of ZDHHC expression on the prognosis of patients with LUAD. A one‐way analysis of variance (ANOVA) test or Student's *t*‐test was used for all other data comparisons using the SPSS software. *p* < 0.05 was considered statistically significant.

## 3 RESULTS

### 3.1 Identifying the prognostic genes of ZDHHCs for LUAD

**FIGURE 1:**
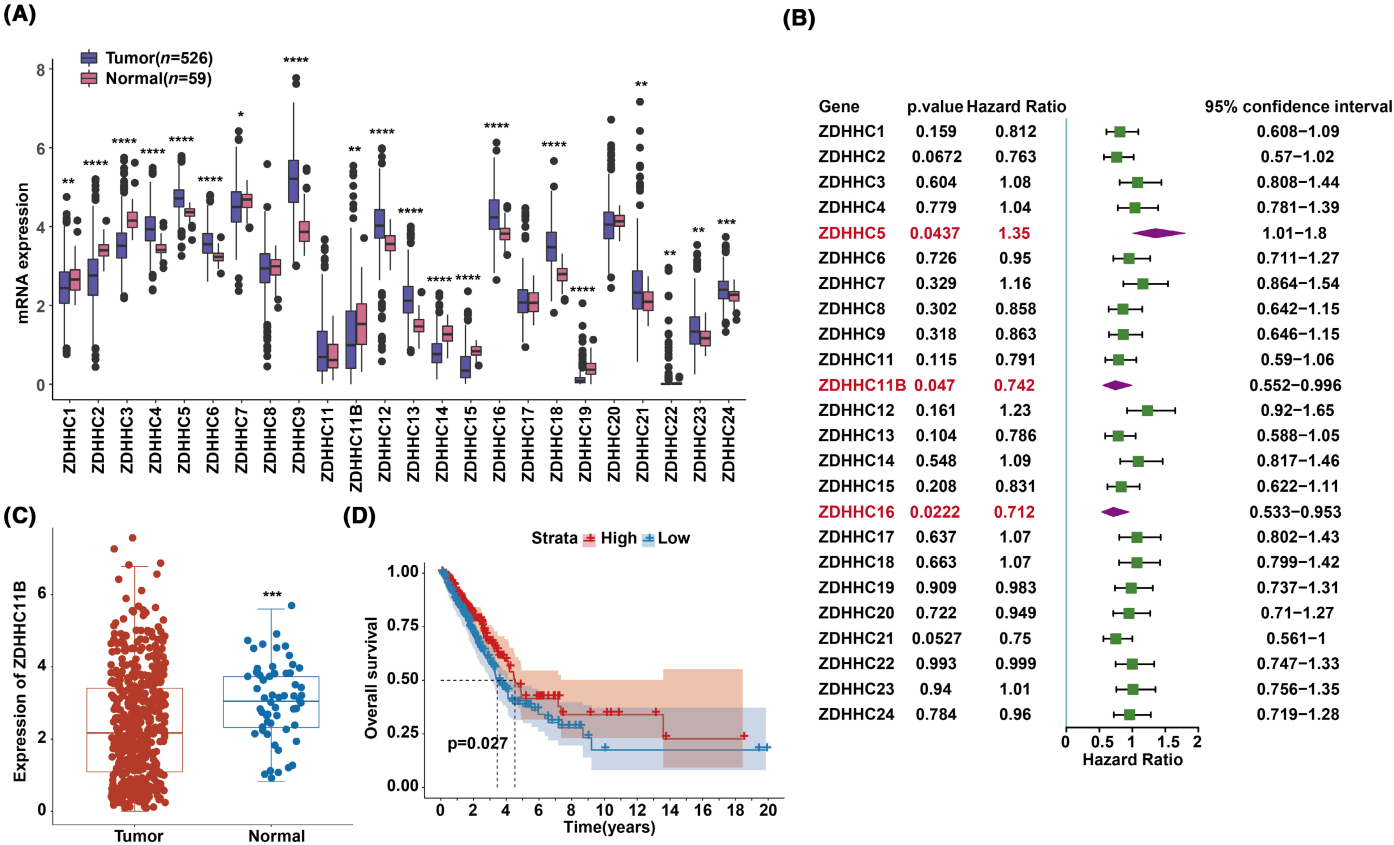
Identifying DEGs and prognostic genes of ZDHHCs in LUAD. (A) DEGs of ZDHHCs in LUAD. (B) Univariate Cox regression analysis of ZDHHCs. (C) Expression level of ZDHHC11B in LUAD. (D) Survival curve of ZDHHC11B in LUAD.

We first investigated the expression patterns of ZDHHCs in LUAD using data from the TCGA database. We found that 20 of the 24 genes exhibited significant differential expression (Figure 1A). Cox regression analysis revealed that ZDHHC5, ZDHHC11B, and ZDHHC16 were significantly associated with survival with a *p*‐value <0.05 (Figure 1B). Among them, the expression level of ZDHHC11B was consistent with prognosis of patients with LUAD. In other words, patients with LUAD with low ZDHHC11B expression have poor prognosis. Subsequently, ZDHHC11B was further evaluated. The mRNA expression level was significantly lower in tumor tissues than in normal tissues as evidenced by TCGA database (Figure 1C). We found that a higher expression of ZDHHC11B led to better survival rates in patients with LUAD (Figure 1D). In summary, ZDHHC11B may act as a tumor suppressor gene in LUAD.

### 3.2 ZDHHC11B downregulation in LUAD

**FIGURE 2:**
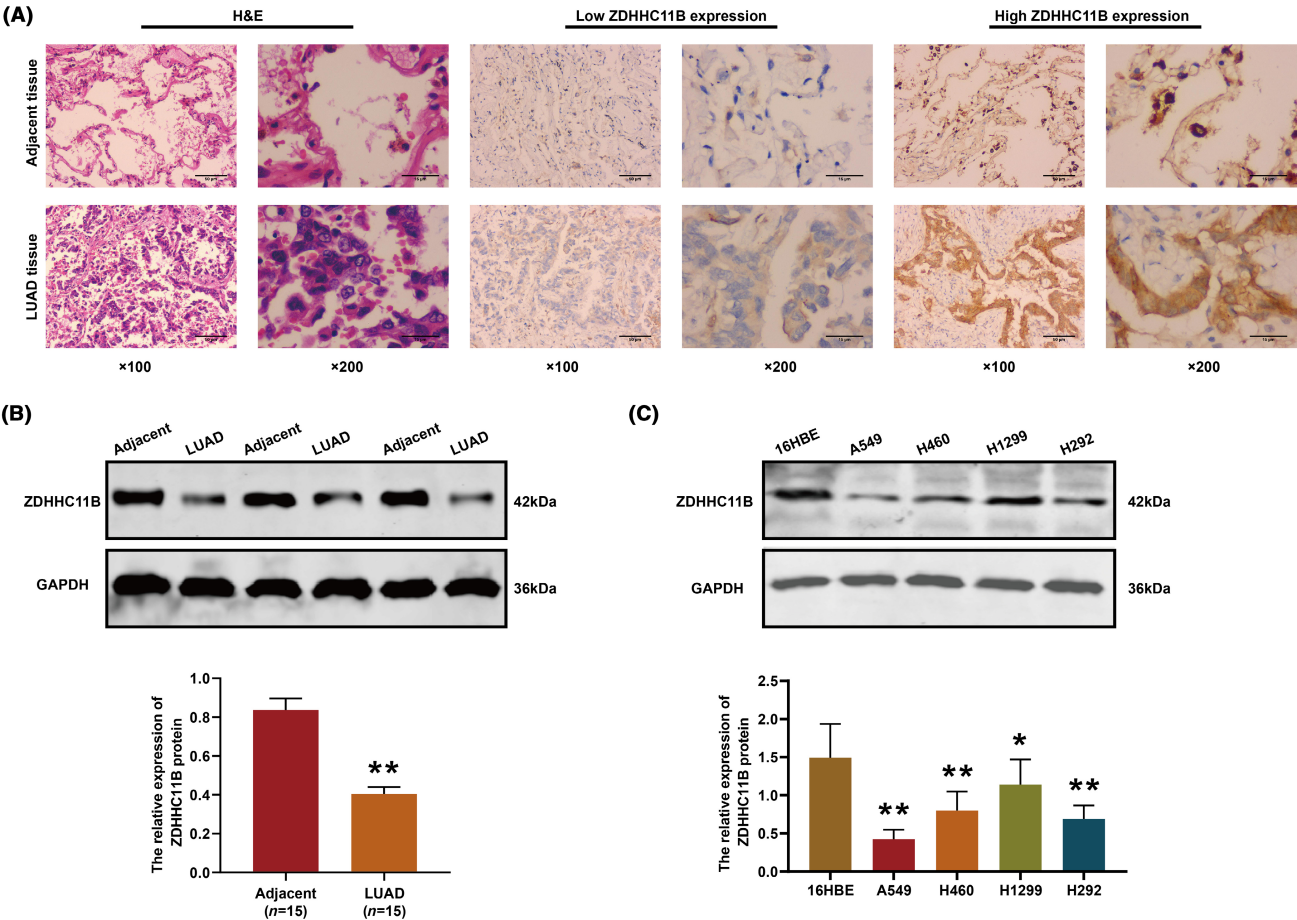
Expression pattern of ZDHHC11B in LUAD tissues and cell lines. (A) ZDHHC11B expression detected using H&E and immunohistochemistry. (B) Protein expression of ZDHHC11B in LUAD tissues detected using western blot. (C) Protein expression of ZDHHC11B in LUAD cells, A549, H460, H1299 H292 and lung epithelial cells 16HBE. Differences were analyzed by the one‐ or two‐way ANOVA followed by Tukey's multiple comparison test. *p > 0.05, **p < 0.001.

Next, we verified the expression pattern of ZDHHC11B in tissues and found that ZDHHC11B was significantly decreased in the LUAD tissues (Figure 2A). Western blot analysis of the LUAD tissues yielded results consistent with those found by immunohistochemistry (Figure 2B). Subsequently, the expression of ZDHHC11B was evaluated in LUAD cells. As shown in Figure 2C, the relative levels of ZDHHC11B in LUAD cells were downregulated, and A549, H292, and H1299 cell lines were chosen for further experiments.

### 3.3 ZDHHC11B inhibited LUAD cell proliferation, migration, and invasion and induced LUAD cell apoptosis in vitro

**FIGURE 3:**
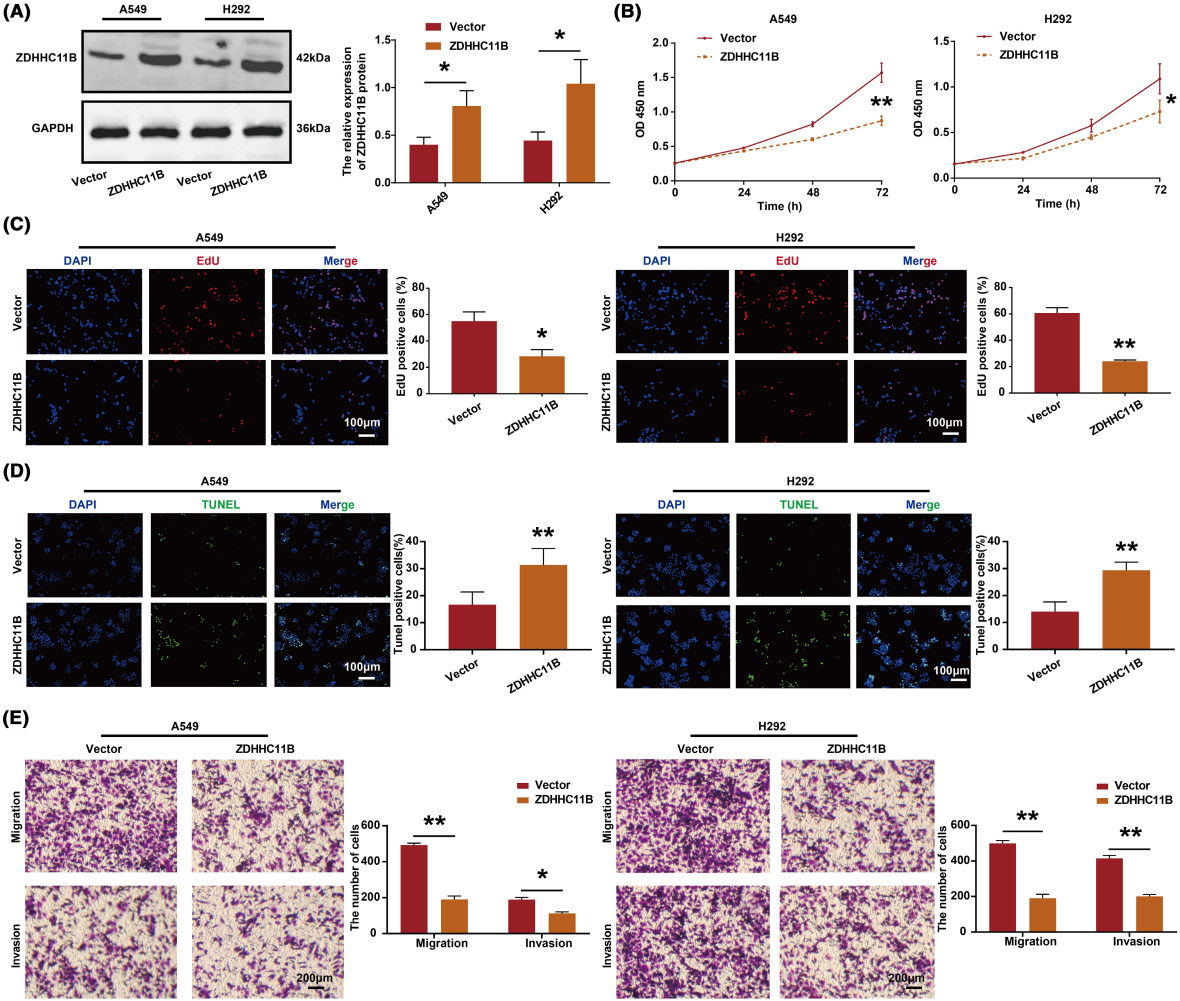
Overexpression of ZDHHC11B inhibited the malignant biological behavior of LUAD cells. (A) Efficiency of ZDHHC11B overexpression was confirmed in A549 and H292 cells. (B) Proliferation of cells was evaluated by CCK‐8 assays. (C) Proliferation of the cells was detected using EdU assays. (D) Apoptosis of the cells was evaluated by TUNEL assays. (E) Migration and invasion of cells were detected using the transwell assay. Differences were analyzed by the one‐ or two‐way ANOVA followed by Tukey's multiple comparison test. *p < 0.05, **p < 0.001.

To evaluate the effect of ZDHHC11B on the malignant biological behavior of LUAD cells in vitro, we overexpressed ZDHHC11B in A549 and H292 cells (Figure 3A). According to the CCK‐8 assay, we found that ZDHHC11B upregulation inhibited cell viability (Figure 3B). The results of the EdU assay showed that the percentage of EdU‐positive cells was reduced in cells overexpressing ZDHHC11B (Figure 3C). According to the data obtained by the TUNEL assays, the percentage of positive cells increased in LUAD cells overexpressing ZDHHC11B (Figure 3D). In addition, migration and invasion were repressed by ZDHHC11B upregulation in transwell assays (Figure 3E).

**FIGURE 4:**
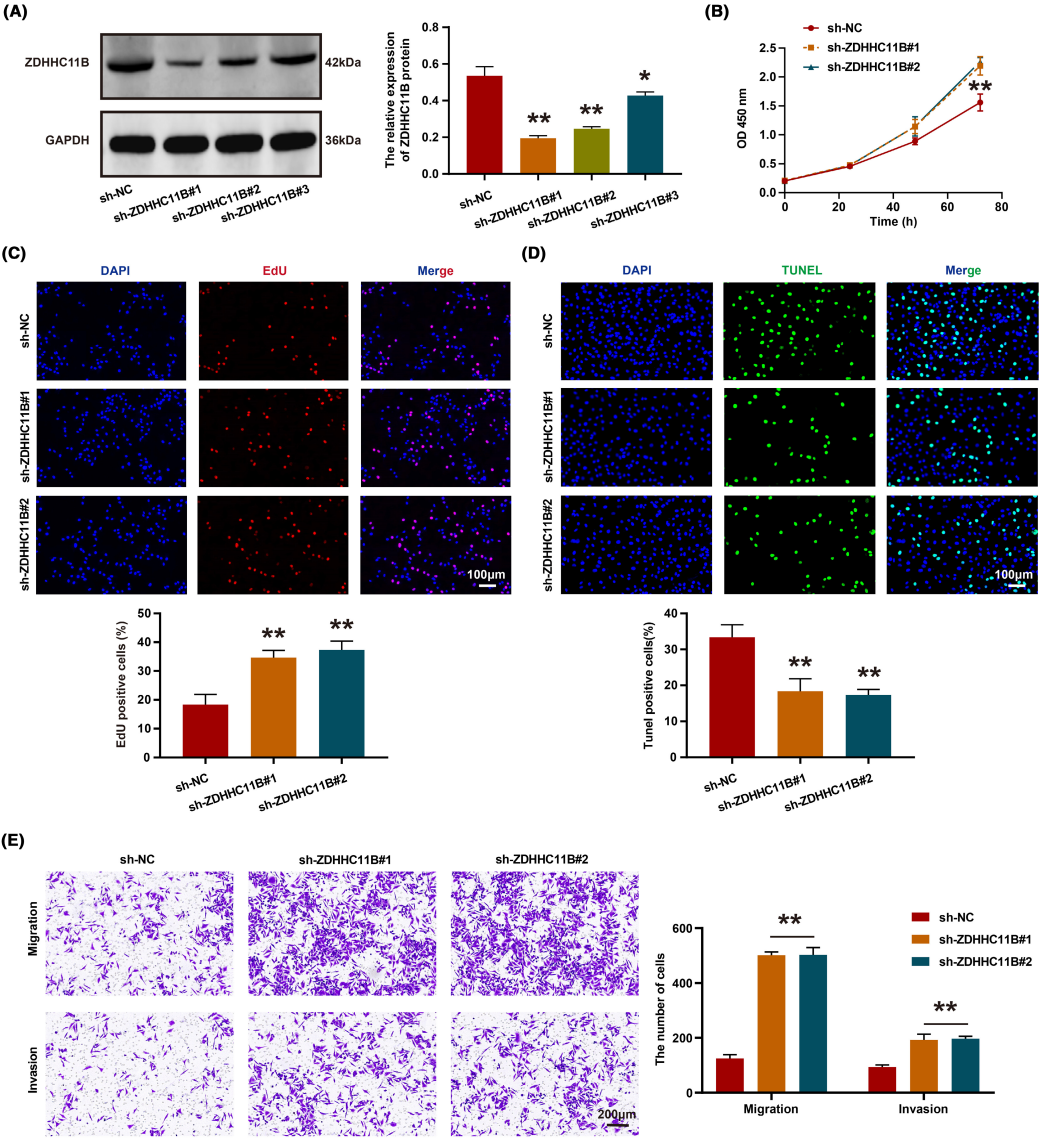
ZDHHC11B knockdown promoted the malignant biological behavior of LUAD cells. (A) Efficiency of ZDHHC11B knockdown was confirmed in H1299 cell lines. (B) Proliferation of cells was evaluated by CCK‐8 assays. (C) Proliferation of cells was detected using EdU assays. (D) Apoptosis of the cells was evaluated by TUNEL assays. (E) Migration and invasion of cells were detected using the transwell assay. Differences were analyzed by the one‐ or two‐way ANOVA followed by Tukey's multiple comparison test. *p > 0.05, **p < 0.001.

Next, we employed ZDHHC11B‐targeting shRNA to knockdown its expression in H1299 cells. Western blot analysis revealed that ZDHHC11B#1 and ZDHHC11B#2 exhibited higher knockdown efficiency (Figure 4A). Subsequently, CCK‐8 and EdU assays were conducted, which demonstrated that the growth and proliferation of H1299 cells were significantly enhanced upon ZDHHC11B knockdown (Figure 4B,C), accompanied by a decrease in the number of apoptotic cells (Figure 4D). Furthermore, transwell assays were performed to assess the effect of ZDHHC11B on the migratory ability of H1299 cells in vitro, revealing that ZDHHC11B knockdown significantly increased the number of cells that migrated or invaded the Transwell chamber (Figure 4E). In summary, ZDHHC11B markedly inhibited the growth, migration, and invasion of LUAD cells in vitro.

### 3.4 ZDHHC11B suppressed LUAD cell growth in vivo

**FIGURE 5:**
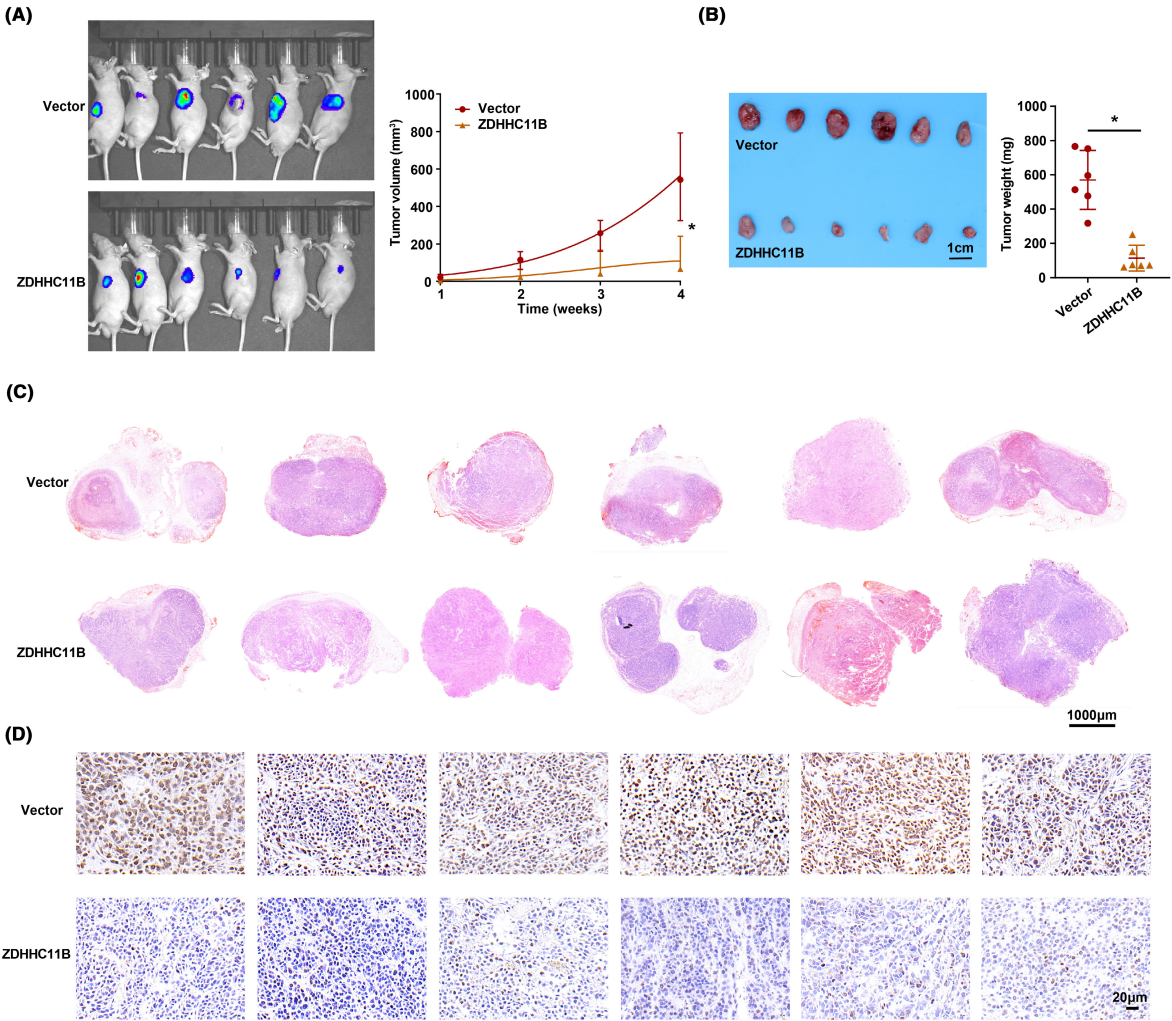
ZDHHC11B suppressed LUAD cell growth in vivo. (A) ZDHHC11B overexpression formed smaller tumors than those in the control group. (B) A xenograft nude model was performed to assess the tumor weight. (C,D) H&E and immunohistochemical staining for ZDHHC11B in xenograft tumors tissues. Differences were analyzed by the one‐ or two‐way ANOVA followed by Tukey's multiple comparison test. *p < 0.001.

To assess the in vivo function of ZDHHC11B, we employed a model of subcutaneous tumor in nude mice for our study. We observed that the rate of tumor growth was slower in the group in which ZDHHC11B was upregulated than in the control group. This was evident from the measurements of tumor volume and weight (Figure 5A,B). Moreover, the expression pattern of ZDHHC11B in tissues was verified by H&E and immunohistochemistry (Figure 5C,D). Therefore, we determined that ZDHHC11B upregulation suppresses LUAD cell growth in vivo.

### 3.5 ZDHHC11B inhibited LUAD progression by regulating epithelial–mesenchymal transition (EMT)

**FIGURE 6:**
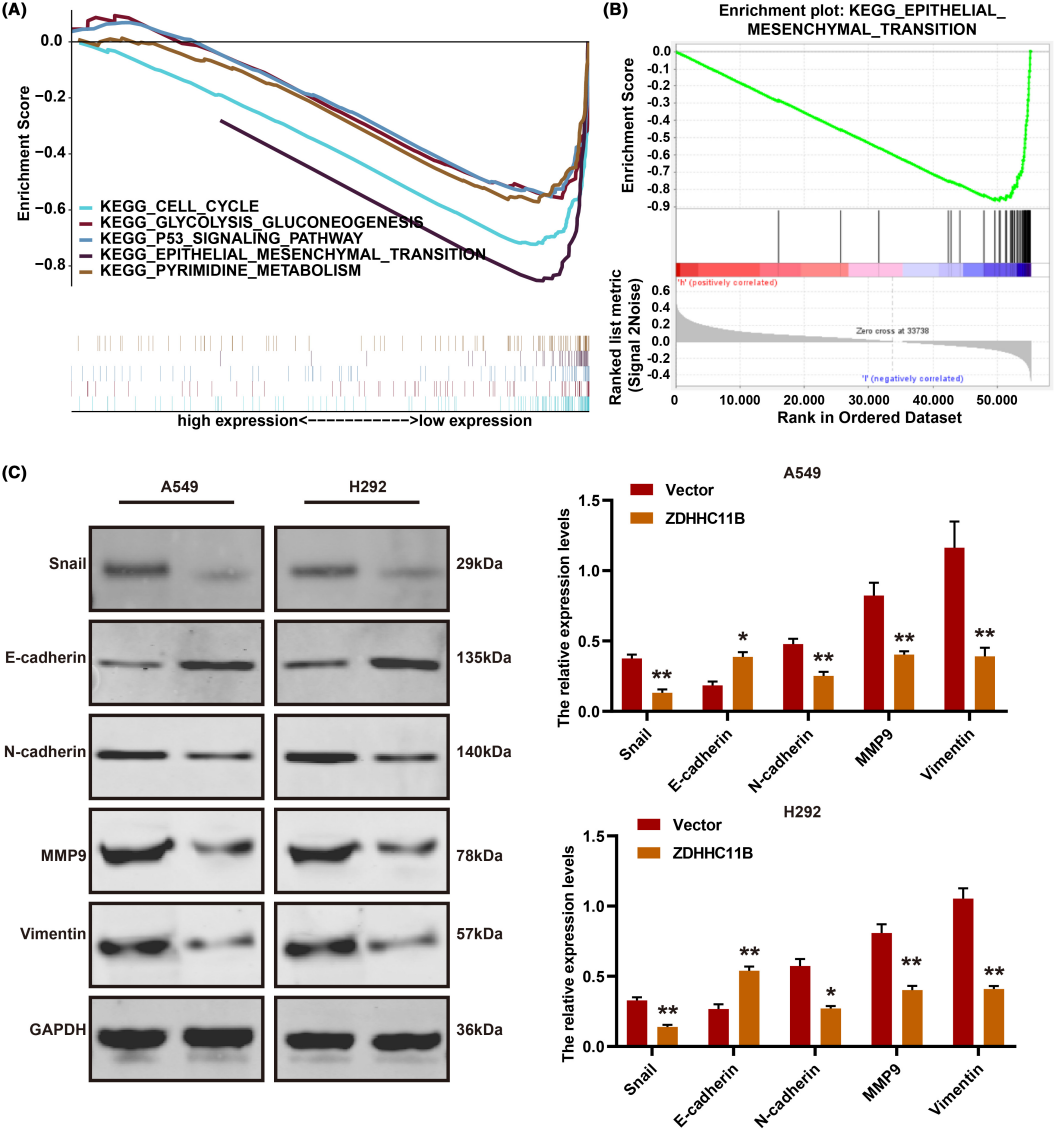
ZDHHC11B regulated EMT. (A) Gene set enrichment analysis related to ZDHHC11B expression in The Cancer Genome Atlas database. (B) ZDHHC11B is closely related to EMT in LUAD. (C) Key factors related to EMT were detected in cells with ZDHHC11B overexpression using western blot. Differences were analyzed by the one‐ or two‐way ANOVA followed by Tukey's multiple comparison test. *p < 0.05, **p < 0.001.

To investigate the potential mechanism of action of ZDHHC11B in LUAD, GSEA was performed. The results demonstrated that ZDHHC11B was positively associated with EMT, PI3K‐AKT, cell cycle, and MAPK pathways (Figure 6A,B). Western blot revealed that ZDHHC11B upregulated EMT by decreasing the expression of several mesenchymal markers, such as Snail, N‐cadherin, vimentin, and MMP9. It also increased the expression of epithelial factors (E‐cadherin) (Figure 6C). These results demonstrated that ZDHHC11B suppressed the migration and invasion of LUAD cells.

## 4 DISCUSSION

LUAD, which arises from small airway epithelial and type II alveolar cells, accounts for approximately 40% of lung cancer cases and is the most frequent type of lung cancer.^18^, ^19^, ^20^ As further research is done on LUAD, molecular targeted therapy is an important treatment for this disease. Target molecules often play an important role in tumor growth and metabolism. It has been found that the epidermal growth factor receptor (EGFR) is associated with cell proliferation and apoptotic functions, and mutations in EGFR often lead to cell tumorigenesis. By contrast, various inhibitors targeting EGFR can effectively keep the proliferation of tumor cells and induce apoptosis.^21^, ^22^ c‐MET is also another important target in LUAD. Its structure is similar to that of EGFR and belongs to receptor tyrosine kinase (RTK). Binding to the ligand hepatocyte growth factor (HGF) leads to conformational changes in c‐MET, activating downstream signaling pathways and participating in the regulation of cell proliferation, invasion, and metastasis.^22^, ^23^ Targeted therapies have driven the development of personalized oncology treatment, but the identified targets are not applicable to all patients with LUAD, and there are still short clinical responses and the emergence of drug‐resistant cells.^24^, ^25^ Therefore, the selection and analysis of target sites remains the next research trend.

The ZDHHC family plays a critical role in post‐translational modification (PTM) within the human body. It is involved in regulating various physiological processes and also plays a significant role in the proliferation and invasion of tumor cells. However, the expression pattern of ZDHHCs was unknown in LUAD. Our study revealed that ZDHHC11B could be a protective factor for LUAD and its low expression was negatively correlated with the prognosis of patients with LUAD. It was found that ZDHHC5, ZDHHC17, ZDHHC18, and ZDHHC23 promote cellular self‐renewal by targeting glioma stem cells, which in turn induces malignant progression of tumor cells.^26^, ^27^, ^28^ We constructed a stable overexpression cell model and conducted functional experiments that identified inhibitory factors for tumor cell growth associated with a high expression of ZDHHC11B. We then conducted tumorigenic experiments in mice that confirmed these results. The ZDHHC family is also an important regulator in LUAD. ZDHHC5 can effectively maintain the proliferative activity of tumor cells and promote cell invasion and metastasis; therefore, increased expression of ZDHHC5 in LUAD tends to predict a poor prognosis.^28^, ^29^ GSEA revealed that ZDHHC11B was mainly enriched in the EMT, and this result was also verified. Palmitoylation is a post‐translational modification that plays a crucial role in regulating protein localization and function, and ZDHHC family members are known to be key regulators of palmitoylation.^30^ There is some evidence suggesting that palmitoylation may play a role in the regulation of EMT markers in cancer. For example, a recent study found that the palmitoylation of E‐cadherin, a key epithelial marker, was necessary for its proper localization and function and that this palmitoylation was regulated by the ZDHHC3 palmitoyltransferase.^31^ Additionally, a study published in 2019 found that the palmitoylation of N‐cadherin, a mesenchymal marker, was required for its stabilization and function in promoting EMT and cancer progression.^32^ However, there is no direct evidence linking ZDHHC11B to the palmitoylation of EMT markers in cancer. Further research is needed to investigate the specific roles of ZDHHC11B and other ZDHHC family members in the regulation of palmitoylation and EMT markers in cancer, as well as their potential as therapeutic targets.

Our study is the first to show that sustained low expression of ZDHHC11B in LUAD reduces the onset of EMT in tumor cells and inhibits tumor growth effectively. Therefore, we speculate that targeting ZDHHC11B may become a potential target for LUAD therapy.

## AUTHOR CONTRIBUTIONS

**Huanyu Dai:** Writing – original draft (equal). **Rui‐Yue Wu:** Methodology (equal). **Jia‐Tong Zhang:** Validation (equal). **Rong Dou:** Validation (equal). **Mao‐Hong Xu:** Validation (equal). **Jia‐Hui Wang:** Validation (equal). **Jun Wang:** Writing – review and editing (equal). **Fei Su:** Project administration (equal). **Tao Zhang:** Writing – review and editing (equal).

## FUNDING INFORMATION

This work was supported by funding from (1) Key research and development plan from Gansu Provincial Department of Science and Technology (22YF7FA086); (2) Natural Science Foundation of Gansu Province (21JR11RA079, 20JR5RA352, 20JR10RA686 and 21JR7RA386); (3) Construction Project of Clinical Medical Research Center from Gansu Provincial Department of Science and Technology (21JR7RA390); (4) Medical Innovation and Development Project of Lanzhou University (lzuyxcx‐2022‐183); (5) Scientific and Technological Development Guiding Plan Project of Lanzhou City (2020‐ZD‐74); (6) The talent Innovation and Entrepreneurship Project of Lanzhou Chengguan District (2021‐9‐17); (7) Student Innovation and Entrepreneurship Action Plan Project of Lanzhou University (2022‐553); (8) Subsidy Project of The First Hospital of Lanzhou University (ldyyn2020‐08, ldyyyn2018‐13, ldyyyn2018‐43, ldyyyn2019‐18 and ldyyyn2019‐19).

## CONFLICT OF INTEREST STATEMENT

The authors declare that the research was conducted in the absence of any commercial or financial relationships that could be construed as a potential conflict of interest.

## ETHICS APPROVAL STATEMENT

The study was approved by the ethics committee of the First Hospital of Lanzhou University.

## Supporting information


Table S1.

## Data Availability

The original contributions presented in the study are included in the article; further inquiries can be directed to the corresponding authors.
