# Supplementary material for: ZDHHC11B is decreased in lung adenocarcinoma and inhibits tumorigenesis via regulating epithelial–mesenchymal transition
Source: Cancer Med. 2023 Jul 11;12(16):17212–22. doi: 10.1002/cam4.6345 (PMC10501301; doi:10.1002/cam4.6345)
Supplement: Supplementary file 1 — Table S1. [file CAM4-12-17212-s001.docx]

Improving Vibrational Spectroscopy Prospects in Frontline Clinical Diagnosis – FTIR on Pancreatic Cancer. Supporting information.

Contents

[**Abstract:** 1](#_Toc136335652)

[1. Pancreatic Patient Information 2](#_Toc136335653)

[2. Methodology and Pre-processing of FTIR spectra 6](#_Toc136335654)

[3. Measuring confidence 9](#_Toc136335656)

[4. Additional validation experiment 9](#_Toc136335657)

[5. Additional table 1 information 11](#_Toc136335658)

# **Abstract:**

The following materials contain additional information not present in the main manuscript for additional clarity and confidence in the results and greater replicability of our methods. Our sample preparation, Instrument specification and statistical methods are equivalent to those in our previous study^1^, with a small iteration on confidence outlined in section 3. For additional confidence, section 4 includes an experiment comparing our Cross-validation with a traditional leave-20%-out method. For additional data that couldn’t be included in the main manuscript, see section 5.

# Pancreatic Patient Information

Table-S1: Table showing patient profiles (anonymous) with the initial diagnosis, final diagnosis, information from ELISA tests and information from our FTIR tests. The general principle for the colour codes: green – predicted correctly with high probability, yellow – predicted correctly with medium probability and red- predicted wrong. More specifically, for Elisa the numbers are a reflection of the concentration of Sial Lewis (Ca-19.9) in the patient’s blood, higher concentrations than 59 U/ml suggesting the patient has pancreatic cancer. More specific ranges of CA19-9 for each diagnostic group (both for ELISA and FTIR) were supplied: 2-30 U/ml classifies benign (Group 3), 33-58 U/ml classifies control (Group 4), 59-81 U/ml classifies early (resectable) cancer (Group 1), and >82 U/ml classifies advanced (severe) stage (Group 2). The same classification has been used for the FTIR diagnosis column. Green indicates the correct diagnosis and patient group. Yellow indicates a sample was correctly diagnosed as cancer/non-cancer but was in the incorrect group. Red indicates incorrect diagnosis. For FTIR the number indicates the certainty of the model’s prediction. Above 0= cancer, below 0 =non-cancer. Yellow indicates that at least one of the 3 repeats was diagnosing incorrectly, but they averaged out to a correct diagnosis. PDAC: pancreatic ductal adenocarcinoma, IPMN: intraductal papillary mucinous neoplasm.

| Initial diagnosis | Final Clinical Diagnosis | Age | Sex | ELISA number | Grouping by ELSIA | FTIR number | Grouping by FTIR |
| --- | --- | --- | --- | --- | --- | --- | --- |
| Group 1, Early Cancer | | | | Correct diagnosis: 1, 2  1-2 | | | |
| 1.03 | Resected PDAC | 73 | M | 110.4 | 2 | 11.0 | 1 |
| 1.04 | Resected PDAC | 62 | M | 136.4 | 2 | 10.1 | 1 |
| 1.05 | Resected PDAC | 61 | M | 30.06 | 3 | 9.8 | 1 |
| 1.08 | Resected PDAC | 70 | M | 143.1 | 2 | 9.6 | 1 |
| 1.12 | Resected PDAC | 68 | M | 19.7 | 3 | 7.5 | 1 |
| 1.14 | Resected PDAC | 71 | M | 19.8 | 3 | 6.3 | 1 |
| 1.16 | Resected PDAC | 56 | M | 88.8 | 2 | 3.4 | 1 |
| 1.17 | Resected PDAC | 69 | M | 151.0 | 2 | 7.0 | 1 |
| 1.18 | Resectable PDAC – Patient borderline fitness – not for surgery | 76 | M | 109.0 | 2 | 4.9 | 1 |
| 1.19 | Resectable PDAC – Delayed due to COVID and larger PE | 65 | M | 129.4 | 2 | 5.4 | 1 |
| 1.20 | Resectable PDAC – Resection delayed due to COVID | 72 | M | 155.0 | 2 | 13.1 | 1 |
| 1.21 | Resectable PDAC – Resection delayed due to COVID | 75 | M | 46.4 | 4 | 4.8 | 1 |
| 1.22 | Resected PDAC | 62 | M | Undetectable | - | 5.2 | 1 |
| Group 2, Late stage Cancer | | | | Correct diagnosis: 1, 2 | | | |
| 1.01 | Metastatic PDAC (Found intra-operatively) | 78 | F | 126.9 | 2 | 6.7 | 1 |
| 1.02 | Locally Advanced PDAC (Found intra-operatively) | 59 | F | 68.4 | 1 | 5.3 | 1 |
| 1.06 | Locally Advanced PDAC (Progressed pre-operatively) | 70 | F | 63.9 | 1 | 13.7 | 1 |
| 1.07 | Locally Advanced PDAC (Progressed pre-operatively) | 67 | M | 125.1 | 2 | 4.2 | 1 |
| 1.09 | Metastatic PDAC (Found intra-operatively) | 74 | M | 111.3 | 2 | 5.5 | 1 |
| 1.10 | Locally Advanced PDAC (Found intra-operatively) | 64 | M | 60.3 | 1 | 7.9 | 1 |
| 1.13 | Metastatic PDAC (Found intra-operatively) | 73 | F | 57.8 | 4 | 5.9 | 1 |
| 1.15 | Metastatic PDAC (Found intra-operatively) | 73 | F | Undetectable | - | -0.2 | 3 |
| 2.01 | Locally Advanced PDAC | 72 | F | 114.6 | 2 | 9.7 | 1 |
| 2.02 | Locally Advanced PDAC | 75 | M | 198.2 | 2 | 4.9 | 1 |
| 2.03 | Locally Advanced PDAC | 72 | F | 175.2 | 2 | 6.0 | 1 |
| 2.04 | Metastatic PDAC | 61 | M | 129.3 | 2 | 9.7 | 1 |
| 2.05 | Metastatic PDAC | 70 | M | 162.9 | 2 | 3.1 | 1 |
| 2.06 | Locally Advanced PDAC | 76 | M | 55.9 | 4 | 5.8 | 1 |
| 2.07 | Locally Advanced PDAC | 69 | F | pending |  | 14.8 | 1 |
| 2.08 | Locally Advanced PDAC | 82 | F | pending |  | 12.9 | 1 |
| 2.09 | Locally Advanced PDAC | 50 | - | pending |  | 13.5 | 1 |
| Group 3, Benign | | | | Correct diagnosis: 3, 4 | | | |
| 3.01 | Chronic Pancreatitis | 45 | F | 94.8 | 2 | -5.2 | 3 |
| 3.02 | Chronic Pancreatitis | 42 | M | 74.9 | 1 | -3.8 | 4 |
| 3.03 | Acute Pancreatitis | 80 | F | 61.2 | 1 | -8.7 | 4 |
| 3.04 | Main Duct IPMN (Cyst) | 72 | F | 4.7 | 3 | -2.9 | 4 |
| 3.05 | Branch Duct IPMN (Cyst) | 68 | F | 43.2 | 4 | -6.3 | 4 |
| 3.06 | Acute Pancreatitis | 68 | M | 23.3 | 3 | -13.2 | 4 |
| 3.07 | Pancreatic Cyst | 51 | M | 73.1 | 1 | -14.6 | 4 |
| 3.08 | Pancreatic Cyst | 34 | F | 18.5 | 3 | -2.9 | 4 |
| 3.11 | Acute Pancreatitis | 80 | F | 15.9 | 3 | -4.1 | 4 |
| 3.12 | IPMN (cyst) | 48 | F | 20.9 | 3 | -1.3 | 4 |
| 3.13 | Acute Pancreatitis | 64 | M | 43.7 | 4 | 0.1 | 2 |
| 3.14 | Acute Pancreatitis | 67 | F | 8.6 | 3 | -7.7 | 4 |
| 3.15 | Chronic Pancreatitis | 40 | F | Undetectable | - | -4.8 | 4 |
| 3.16 | Chronic Pancreatitis | 52 | M | 17.8 | 3 | -4.5 | 4 |
| 3.17 | Acute Pancreatitis | 70 | F | 40.6 | 4 | -6.6 | 4 |
| 3.18 | Acute Pancreatitis | 69 | M | Undetectable | - | -5.0 | 4 |
| 3.19 | Acute Pancreatitis | 59 | F | 42.9 | 4 | -6.5 | 4 |
| 3.20 | Acute Pancreatitis | 50 | F | 34.8 | 3 | -11.9 | 4 |
| 3.21 | Acute Pancreatitis | 43 | M | 10.4 | 3 | -10.4 | 4 |
| 3.22 | Chronic Pancreatitis | 68 | M | 46.9 | 4 | -10.2 | 4 |
| 3.23 | Acute Pancreatitis | 42 | M | 36.1 | 3 | -9.1 | 4 |
| 3.24 | Chronic Pancreatitis | 60 | M | 10.6 | 3 | -13.8 | 4 |
| 3.25 | Acute Pancreatitis | 44 | M | 43.7 | 4 | -6.6 | 4 |
| 3.26 | Pancreatic Cyst | 69 | F | 19.1 | 3 | -8.5 | 4 |
| 3.27 | Acute Pancreatitis | 55 | F | 15.4 | 3 | -4.4 | 4 |
| 3.28 | Acute Pancreatitis | 86 | F | 15.4 | 3 | -4.1 | 4 |
| 3.29 | Cyst IPMN | 83 | F | 10.6 | 3 | -9.0 | 4 |
| 3.30 | Chronic Pancreatitis | 65 | F | 40.6 | 3 | -14.0 | 4 |
| 3.32 | Branch Duct IPMN | 76 | F | 33.7 | 3 | -5.8 | 4 |
| 3.33 | IPMN (Cyst) | 64 | F | 36.6 | 3 | -13.0 | 4 |
| Group 4, Control | | | | Correct diagnosis: 3, 4 | | | |
| 1.11 | Benign Biliary Stricture (initially presumed to be PDAC) | 74 | F | 25.7 | 1 | -9.3 | 4 |
| 3.09 | CDKN2A mutation | 56 | F | 10.3 | 3 | -12.5 | 4 |
| 3.10 | CDKN2A mutation | 63 | F | 32.4 | 3 | -1.7 | 3 |
| 4.01 | Gallstones | 65 | M | 23.1 | 3 | -13.6 | 4 |
| 4.02 | Gallstones | 60 | F | 55.8 | 4 | -6.5 | 4 |
| 4.03 | Gallbladder Adenomyosis | 57 | F | 152.5 | 2 | -7.3 | 4 |
| 4.04 | Anal Fistula | 47 | F | 16.4 | 3 | -11.5 | 4 |
| 4.05 | Incisional Hernia | 72 | M | 42.5 | 4 | -8.1 | 4 |
| 4.06 | Inguinal Hernia | 67 | M | 14.8 | 3 | -7.9 | 4 |
| 4.07 | Sleeve Gastrectomy (Bariatric) | 60 | F | Undetectable | - | -10.4 | 4 |
| 4.08 | Gallstones | 59 | F | 9.2 | 3 | -9.3 | 4 |
| 4.09 | Umbilical Hernia | 56 | F | 8.4 | 3 | -12.5 | 4 |

# Methodology and Pre-processing of FTIR spectra


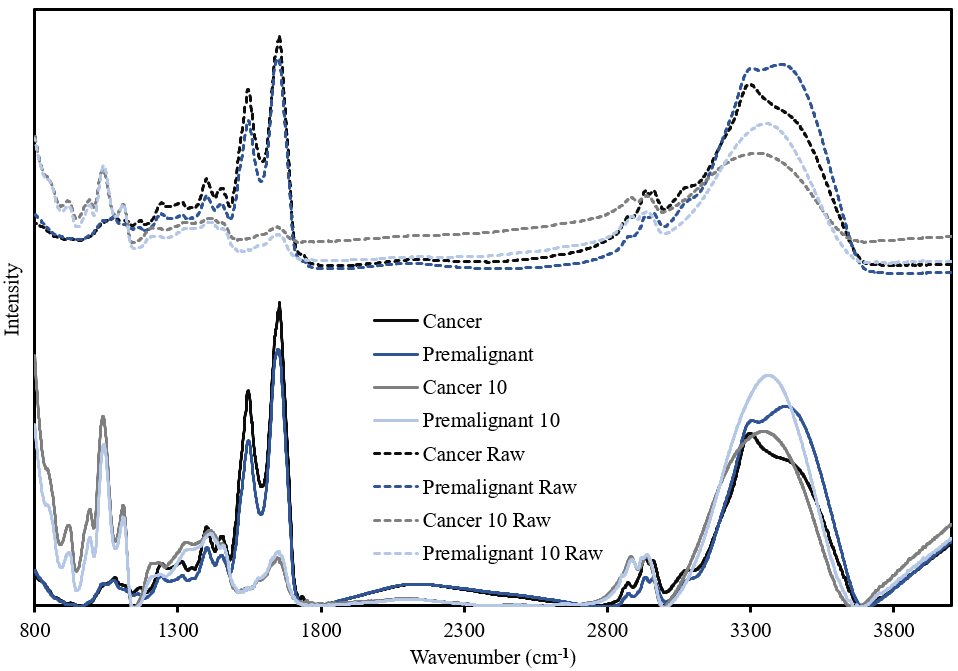
The details for the methodology are broadly equivalent to those elaborated on in Duckworth et al. (2022)^1^. A few small alterations, e.g. changing the confidence interval calculation to one that better handles extremes and some additional elaboration on the methods are presented here.

Figure-S1: Transmission mode FTIR spectra before (raw, dotted) and after baselining and normalisation. A set of healthy cancerous and premalignant samples were used for this example, showing both whole serum and <10 kDa subsets.

Spectra were pre-processed with a background correction using the asymmetric least squares smoothing (ALSS) method. The method uses a smoothing algorithm with an asymmetric weighting of deviations to get a baseline estimator. This allows a corrective baseline to be quickly obtained while retaining the signal peak information. Baselining was followed by average normalisation by dividing by the average intensity for each spectrum. Spectra were also trimmed to 800-1800 cm^-1^ to focus on the fingerprint region (internal testing produced more efficient classification when doing so) and to remove some obstructive noise between 750-800 cm^-1^.

##
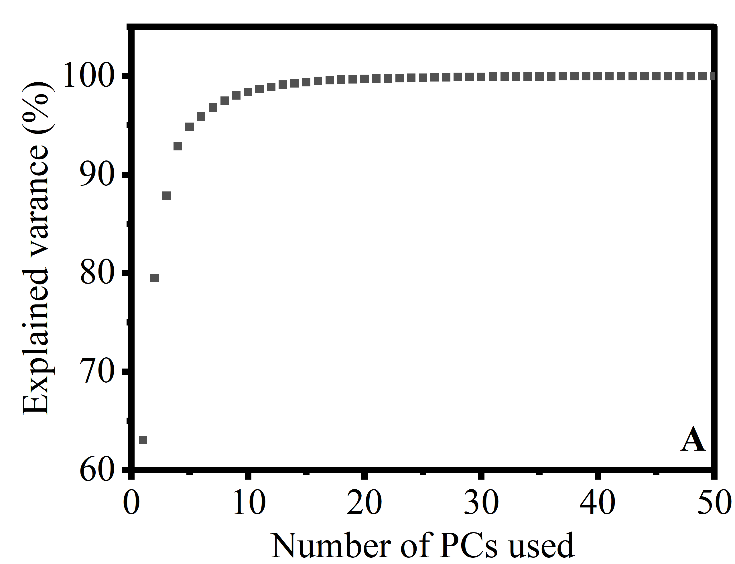

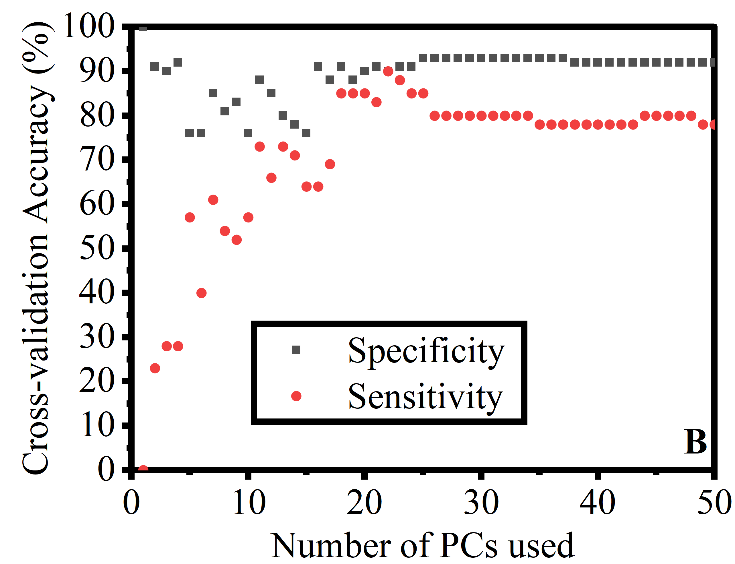


Figure-S2: A) Explained variance graph depending on the number of principal components (PCs) used. B) Cross-validated sensitivity and specificity values are dependent on the number of PCs used in the model. Example graph to demonstrate how the accuracy plateaus after a certain number of principal components. Before the plateau, the classification is not yet optimised, resulting in lower accuracy. The cross-validated accuracy does not decrease after a point as the SVM algorithm ignores the unnecessary components and minimal or no overfitting occurs. The two graphs mimic one another, the plateau in B starts at 20 principle components where in A there is 99.7% variance explained. This example is for classifying <10kDa Premalignant v Cancer.

|  |  |
| --- | --- |
| 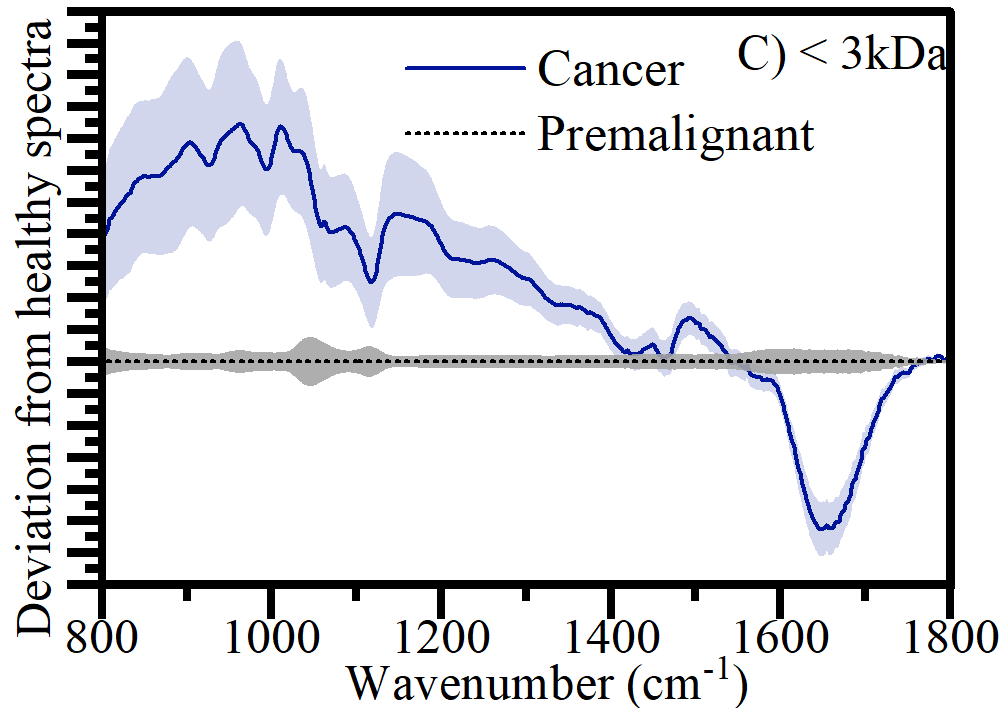  A) | 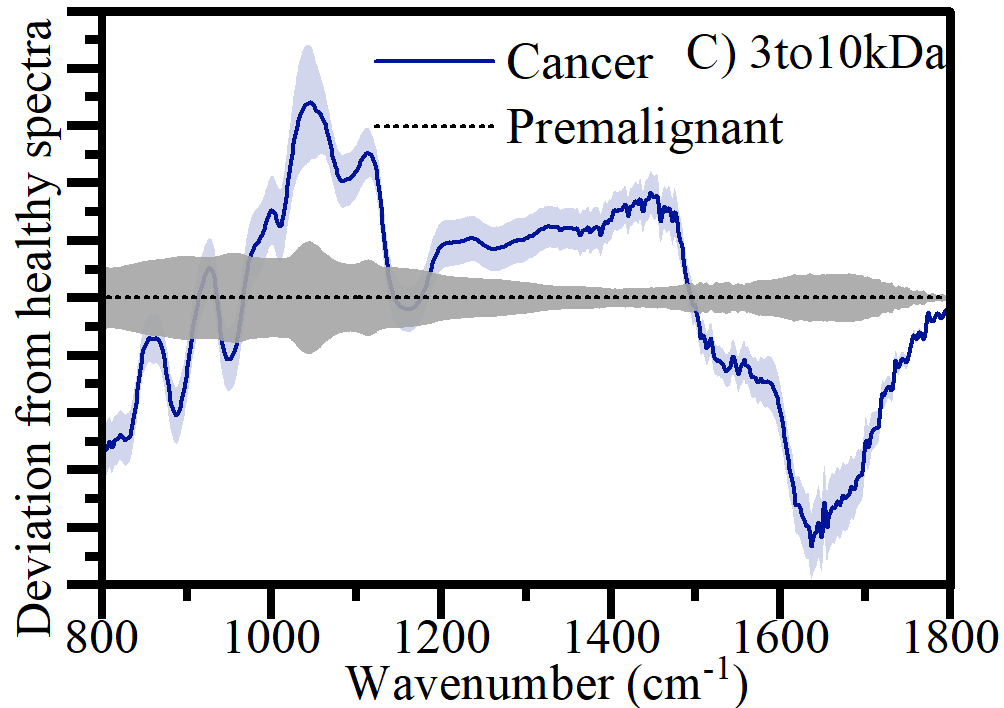  B) |

Figure S3: Difference in the average spectra of cancer and premalignant patient serum from healthy in the small-cohort windowing experiment. (A) <3 kDa and (B) 3-10 kDa window. The faded colour around each line shows the spread for different patients.

# Measuring confidence

Confidence intervals are a suitable method for assessing confidence in a particular classification method’s accuracy. For our purposes, 95% confidence is typically recommended, though there may be situations where either less is sufficient or higher confidence is required – e.g. to avoid potential extreme negative outcomes.

The ‘Clopper Pearson’s interval, sometimes termed exact interval, is a method that helps address this inconsistency at extreme predictive values^2^. It uses the inverse of the cumulative beta probability density function to produce a similar confidence value to the Wald interval but with more consistency at the extremes. The interval produced also will typically have different positive and negative values. For example, a 100% accurate classifier for 50 cancer and 50 healthy patients would have a 95% confidence interval between 96.4 and 100%.

| Table-S2: confusion matrix for the LOO cross-validation for <10 kDa plasma, Cancer v Premalignant, with 19% of data removed for an additional validation set. Figures are displayed model/validation. | | | | |
| --- | --- | --- | --- | --- |
| Model/Validation:  Number of spectra = 171/39 (81/19%) | | True clinical diagnosis | |  |
|  |  | Cancer | Premalignant | Total: |
| Model predicted  diagnosis | Cancer | True Positive:  65/14 | False Positive:  10/7 | 75/21 |
|  | Premalignant | False Negative:  13/1 | True Negative:  83/17 | 96/18 |
|  | Total: | 75/15 | 96/24 | Accuracy: **86/86%**  Confidence: 5% |

# Additional validation experiment

Validation of this methodology can be seen in Table S3, where the PCA-SVM + leave-one-out cross-validation process is used to produce a model on 81% of the patients. The accuracy is slightly lower than the full cohort due to the reduced sample size. The produced cross-validation accuracy is proven to correspond to the accuracy for using the model to classify the 19% left-out data. The 86% accuracy validation is equal to and, therefore comfortably within the confidence of, the 86% accurate model.

# Additional Table 1 information

| Table-S3: Additional information about the results in Table-1 in the manuscript, that couldn’t be fit into the main table in the manuscript. Accuracies are all post-cross-validation. | | | | | | |
| --- | --- | --- | --- | --- | --- | --- |
| Fraction | Subsets compared | PCs used | PCA-SVM Acc. (%) | 95% Confidence interval(%) | SVM only Acc. (%) | 95% Confidence interval(%) |
| Whole serum | C v P | 33 | 84.3 | 77.3-90.4 | 75.3 | 67.4-82.1 |
|  | C v H | 10 | 100.0 | 95.6-100.0 | 92.4 | 84.3-97.1 |
|  | C+EC v H+P | 17 | 81.3 | 85.7-94.2 | 75.7 | 69.4-81.2 |
|  | C v EC | 20 | 83.5 | 80.6-95.8 | 76.9 | 67.0-85.0 |
| <10kDa window | C v P | 22 | 90.0 | 84.5-95.1 | 86.5 | 79.4-91.7 |
|  | C v H | 8 | 95.3 | 87.8-98.97 | 87.2 | 77.1-93.9 |
|  | C+EC v H+P | 20 | 90.6 | 85.7-99.0 | 89.2 | 84.0-93.1 |
|  | C v EC | 19 | 90.0 | 80.6-95.8 | 58.6 | 46.3-70.1 |

**References:**

(1) Duckworth, E.; Hole, A.; Deshmukh, A.; Chaturvedi, P.; Chilakapati, M. K.; Mora, B.; Roy, D. Improving Vibrational Spectroscopy Prospects in Frontline Clinical Diagnosis: Fourier Transform Infrared on Buccal Mucosa Cancer. Analytical Chemistry 2022. DOI: 10.1021/acs.analchem.2c02496.

(2) Borek Puza and Terence, O. n. Generalised Clopper–Pearson confidence intervals for the binomial proportion. Journal of Statistical Computation and Simulation 2006, 76 (6), 489-508. DOI: 10.1080/10629360500107527.
